# Microglial gene signature reveals loss of homeostatic microglia associated with neurodegeneration of Alzheimer’s disease

**DOI:** 10.1186/s40478-020-01099-x

**Published:** 2021-01-05

**Authors:** Akira Sobue, Okiru Komine, Yuichiro Hara, Fumito Endo, Hiroyuki Mizoguchi, Seiji Watanabe, Shigeo Murayama, Takashi Saito, Takaomi C. Saido, Naruhiko Sahara, Makoto Higuchi, Tomoo Ogi, Koji Yamanaka

**Affiliations:** 1 Department of Neuroscience and Pathobiology, Research Institute of Environmental Medicine, Nagoya University, Aichi, 464-8601 Japan; 2 Department of Neuroscience and Pathobiology, Nagoya University Graduate School of Medicine, Aichi, 466-8550 Japan; 3 Department of Genetics, Research Institute of Environmental Medicine, Nagoya University, Aichi, 464-8601 Japan; 4 Department of Human Genetics and Molecular Biology, Nagoya University Graduate School of Medicine, Aichi, 466-8550 Japan; 5 Research Center for Genome and Medical Sciences, Tokyo Metropolitan Institute of Medical Science, Tokyo, 156-8506 Japan; 6 Research Center for Next-Generation Drug Development, Research Institute of Environmental Medicine, Nagoya University, Aichi, 464-8601 Japan; 7 Department of Neuropsychopharmacology and Hospital Pharmacy, Nagoya University Graduate School of Medicine, Aichi, 466-8550 Japan; 8 Brain Bank for Aging Research, Tokyo Metropolitan Geriatric Hospital and Institute of Gerontology, Tokyo, 173-0015 Japan; 9 Brain Bank for Neurodevelopmental, Neurological and Psychiatric Disorders, United Graduate School of Child Development, Osaka University, Osaka, Japan; 10 Department of Neurocognitive Science, Institute of Brain Science, Nagoya City University Graduate School of Medical Sciences, Aichi, 467-8601 Japan; 11 Laboratory for Proteolytic Neuroscience, RIKEN Center for Brain Science, Saitama, 351-0198 Japan; 12 Department of Functional Brain Imaging, National Institute of Radiological Sciences, National Institutes for Quantum and Radiological Science and Technology, Chiba, 263-8555 Japan

**Keywords:** Alzheimer’s disease, Animal model, Next generation sequence, Microglia, Precuneus, Neuroinflammation

## Abstract

Microglia-mediated neuroinflammation has been implicated in the pathogenesis of Alzheimer’s disease (AD). Although microglia in aging and neurodegenerative disease model mice show a loss of homeostatic phenotype and activation of disease-associated microglia (DAM), a correlation between those phenotypes and the degree of neuronal cell loss has not been clarified. In this study, we performed RNA sequencing of microglia isolated from three representative neurodegenerative mouse models, *App*^*NL*-*G*-*F/NL*-*G*-*F*^ with amyloid pathology, rTg4510 with tauopathy, and SOD1^G93A^ with motor neuron disease by magnetic activated cell sorting. In parallel, gene expression patterns of the human precuneus with early Alzheimer’s change (n = 11) and control brain (n = 14) were also analyzed by RNA sequencing. We found that a substantial reduction of homeostatic microglial genes in rTg4510 and SOD1^G93A^ microglia, whereas DAM genes were uniformly upregulated in all mouse models. The reduction of homeostatic microglial genes was correlated with the degree of neuronal cell loss. In human precuneus with early AD pathology, reduced expression of genes related to microglia- and oligodendrocyte-specific markers was observed, although the expression of DAM genes was not upregulated. Our results implicate a loss of homeostatic microglial function in the progression of AD and other neurodegenerative diseases. Moreover, analyses of human precuneus also suggest loss of microglia and oligodendrocyte functions induced by early amyloid pathology in human.

**Supplementry information:**

The online version of this article (10.1186/s40478-020-01099-x) contains supplementary material, which is available to authorized users.

## Introduction

Alzheimer’s disease (AD) is the most common neurodegenerative disease that causes dementia, neuropathologically characterized by the accumulation of amyloid β (Aβ), phosphorylated Tau, and neuronal dystrophy and loss [33]. Neuroinflammation is defined as an inflammatory response within the central nervous system (CNS), and it is mediated by activation of the innate immune system of the brain in response to inflammatory challenges, including misfolded protein aggregates that often accumulate in lesions of neurodegenerative diseases [22, 43]. Microglia are the resident innate immune cells of the CNS, and are key players to mediate neuroinflammation, playing critical roles in the recognition and clearance of Aβ in AD [9, 15, 27]. The activation phenotype of microglia was previously classified by the expression pattern of cytokines in analogy of activated macrophages: the proinflammatory “classical” activation phenotype (M1) and the anti-inflammatory “alternative” activated phenotype (M2) [44]. However, this simplistic view of microglial phenotypes does not adequately reflect the complex physiology of microglia [35]. The progression of neurodegenerative disease induces the loss of microglial homeostatic molecules and functions [16], leading to chronically progressive neuroinflammation [31]. In addition, recent studies demonstrated that a common disease-associated microglia (DAM) or “neurodegenerative” phenotype, defined by a small set of upregulated genes, was observed in neurodegenerative diseases including AD, amyotrophic lateral sclerosis (ALS), and frontotemporal dementia, and aging [10, 20, 24]. However, it remains unclear whether the loss of homeostatic function in microglia or the DAM phenotype is correlated with the degree of neuronal cell loss, and whether DAM is beneficial or detrimental to neurodegenerative diseases.

Several transcriptomic studies using single nucleus analysis examined the molecular aspects of neuroinflammation in the prefrontal or entorhinal cortex of AD [11, 26, 49]; however, neuroinflammatory alteration in the precuneus, which is vulnerable to Aβ deposition in preclinical AD, has yet to be examined. The precuneus is medially located in the parietal lobe of the cerebral cortex, and it is a component of the default mode network, which is implicated in episodic memory retrieval, and displays high metabolic activity during the baseline resting state [6]. Amyloid PET studies demonstrated that the precuneus is one of the brain regions where Aβ accumulation preferentially starts in preclinical AD [30, 47]. Therefore, it is important to uncover the neuroinflammatory aspects of the precuneus at the early amyloid pathology stage to better understand microglial response in early AD.

In this study, we first performed comparative gene expression analysis of isolated microglia from the three mouse models of neurodegenerative diseases: *App*^*NL*-*G*-*F/NL*-*G*-*F*^ mice that display an amyloid pathology [37], rTg4510 mice with tauopathy [38], and SOD1^G93A^ mice with motor neurodegeneration [12]. Despite robust neuroinflammation with microglial responses in all mouse models, *App*^*NL*-*G*-*F/NL*-*G*-*F*^ mice do not show neuronal death, whereas rTg4510 and SOD1^G93A^ mice show a substantial loss of neurons. We found that most homeostatic microglial genes were downregulated in rTg4510 and SOD1^G93A^ mice, and were correlated with the degree of neuronal cell loss. In contrast, DAM genes were uniformly upregulated in all disease models, and this alteration was not correlated with neurodegeneration. Moreover, precunei of individuals with early AD pathology show downregulation of some microglial genes linked to homeostatic functions and genes linked to oligodendrocyte function.

## Materials and methods

### Postmortem human brain tissues

The postmortem brains from 25 individuals (non-AD = 14 and AD = 11) were obtained by autopsy with informed consent and diagnosed by a neuropathologist in the Brain Bank for Aging Research, Tokyo Metropolitan Geriatric Hospital and Institute of Gerontology. All subjects with early AD pathology did not have apparent family history of dementia, therefore, were recognized as sporadic cases. The subjects were neuropathologically grouped according to the neurofibrillary tangle staging of Braak and Braak [2]. The tissues were dissected from precuneus. A variation in the ratio of gray and white matter of dissected precuneus tissues, which may cause a substantial effect on relative levels of oligodendrocyte-derived mRNAs, was negligible, since we confirmed there was no changes in the *OLIG1/2* mRNA levels in non-AD and AD brain as internal controls. The use of human postmortem brain tissue was approved by the Ethics Committee of Research Institute of Environmental Medicine, Nagoya University and Tokyo Metropolitan Institute. Brain tissues for RNA preparation were immediately frozen using liquid nitrogen and stored at − 80 °C before use.

### Animals

Heterozygous *App*^+*/NL*-*G*-*F*^ mice (C57BL/6-App < tm3(NL-G-F)Tcs >), carrying *App* gene with humanized Aβ sequence (G676R, F681Y, R684H), Swedish (KM670/671NL), Beyreuther/Iberian (I716F), and Arctic (E693G) mutations, were previously established by a knock-in strategy [37]. Homozygous *App*^*NL*-*G*-*F/NL*-*G*-*F*^ and wild-type mice were obtained by crossbreeding, and were maintained as inbred lines. Tg(Camk2a-tTA)1Mmay *Fgf14*^*Tg(tetO*-MAPT*P301L)4510Kha^/J mice were previously established [38]. A parental mutant tau responder line, conditionally expressing the 4R0N isoform of human P301L mutant tau, in the FVB/N strain (Clea Inc., Tokyo, Japan), and a tTA activator line, under the control of CaMKII promoter, in the 129 + ter/SV strain (Clea Inc.) were crossbred to generate rTg4510 mice [18]. tTA activator line was used as control for rTg4510 mice. Transgenic mice expressing the inherited ALS-linked human SOD1^G93A^ gene (B6.Cg-Tg (SOD1*G93A)1Gur/J) on the C57BL/6 background were obtained from Jackson Laboratory (Bar Harbor, ME, USA) [12]. Genotyping of mice was performed as previously described [18, 23, 37].

All mice were maintained under a standard specific pathogen-free environment (12 h light–dark-cycle; 23 ± 1 °C; 50 ± 5% humidity) with free access to food and water throughout experiments. Animals were treated in compliance with the guidelines established by the Institutional Animal Care and Use Committee of Nagoya University and National Institutes for Quantum and Radiological Science and Technology.

### Microglia isolation from brain and spinal cord

Magnetic-activated cell sorting (MACS) of microglia in brain or spinal cord is performed as described elsewhere [23]. In brief, the cerebral cortex or spinal cord, dissected from mice transcardially perfused with phosphate-buffered saline (PBS), was dissociated at 37 °C for 15 min using the Neural Tissue Dissociation Kit-Postnatal Neurons (Miltenyi Biotec, Bergisch-Gladbach, Germany) by the gentle MACS Dissociator (Miltenyi Biotec). For isolation of microglia, myelin debris was removed by using Myelin Removal Beads II (Miltenyi Biotec). Purified cells were incubated with anti-CD16/CD32 antibodies (Thermo Fisher Scientific, Waltham, MA, USA) for blocking Fc receptors, and then incubated with anti-CD11b microBeads (Miltenyi Biotec) for isolating microglia. CD11b-positive microglia were isolated by magnetic cell sorting through an LS column (Miltenyi Biotec).

### RNA-seq experiments

For the RNA-seq of the mouse samples, total RNA was extracted from MACS-isolated microglia of each models using an RNeasy Mini Kit (Qiagen, Hilden, Germany). The RNAs were sampled from cerebral cortices of 8-month-old *App*^*NL*-*G*-*F/NL*-*G*-*F*^ and 7-month-old rTg4510 mice and lumbar spinal cords of 5-month-old SOD^G93A^ mice together with the corresponding wild-type or control mice, respectively. For the RNA-seq of the human samples, total RNA was prepared from precuneus of frozen postmortem brain using mirVana™ miRNA Isolation Kit (Thermo Fisher Scientific, Waltham, MA, USA) according to the manufacturer instructions. The total RNA was qualified by using Agilent 2100 Bioanalyzer (Agilent Technologies, Santa Clara, CA, USA). Libraries were prepared by using TruSeq mRNA or TruSeq Stranded mRNA (Illumina, San Diego, CA, USA), and, from these libraries, 151-nt paired-end reads were sequenced on the HiSeq X Ten with the HiSeq X Reagent Kits (Illumina) and the NovaSeq 6000 with the NovaSeq Reagent Kits (Illumina).

### In silico analysis of the RNA-seq data

The reference genome assemblies and gene annotations, the mouse genome version UCSC mm10 and the human genome version UCSC hg38, were retrieved from iGenomes (https://support.illumina.com/sequencing/sequencing_software/igenome.html). The Illumina adapter sequences, as well as low quality bases (quality score < 20), were trimmed from 3′-ends of the sequencing reads with the Trim Galore v0.5.0 (https://www.bioinformatics.babraham.ac.uk/projects/trim_galore/). The reads were qualified with FastQC v0.11.8 (https://www.bioinformatics.babraham.ac.uk/projects/fastqc/) before and after trimming. The processed reads were mapped on the reference genome assembly with HISAT2 v2.1.0 [21]. Mapping reads prepared with the stranded kit was processed by employing strand specificity information. The alignments on rDNA regions were removed with BEDTools v2.25.0 [34]. From mapping data, expressions of the individual annotated genes were quantified with StringTie v1.3.5 [32]. Heatmaps of the gene expression profiles of target gene sets were created with the pheatmap package implemented in R (https://cran.r-project.org/web/packages/pheatmap/).

Differential expression analysis was performed using the Gene Spring Ver. 14.9.1 software from Agilent Technologies (Santa Clara, CA, USA) according to the manufacture’s protocol. Relative similarities of gene expression profiles of the samples were represented by a plot of the principal component analysis (PCA). The PCA analysis was performed with the prcomp function in R by using CPM values of the individual genes.

### Quantification of mRNA levels by real-time PCR

Total RNA was extracted from MACS-sorted microglia using the RNeasy Micro Kit (Qiagen) according to the manufacturer’s instructions. Complementary DNA (cDNA) from MACS sorted cells was generated and amplified from 2.5 or 5 ng of total RNA by using the PrimeScript™ RT reagent Kit (Perfect Real Time) (TaKaRa Bio, Kusatsu, Japan) and 1/50 of the yield was amplified with the SYBR Premix Ex Taq II (Tli RNaseH Plus) (TaKaRa Bio) using the Thermal Cycler Dice Real Time System II or III (TaKaRa Bio). The thermocycle protocol was as follows: 1 cycle at 95 °C for 30 s, 40 cycles at 95 °C for 5 s and 60 °C for 30 s, and a dissociation stage of 95 °C for 15 s, 60 °C for 30 s, and 95 °C for 15 s. Actin was used for normalization. The primers used for real-time RT-PCR were as follows: human *ACTB* primer, forward, 5′-CTGGAACGGTGAAGGTGACA-3′; reverse, 5′-CGGCCACATTGTGAACTTTG-3′; human *HBEGF* primer, forward, 5′-GGACCCATGTCTTCGGAAAT-3′; reverse, 5′-CCCATGACACCTCTCTCCAT-3′; mouse *Actb* primer, forward, 5′-CGGACTCATCGTACTCCTGCTT-3′; reverse, 5′-TTGGCCTCACTGTCCACCTT-3′; mouse *P2ry12* primer, forward, 5′-CATTGACCGCTACCTGAAGACC-3′; reverse, 5′-GCCTCCTGTTGGTGAGAATCATG-3′; mouse *Sall1* primer, forward, 5′-TGTCAAGTTCCCAGAAATGTTCCA-3′; reverse, 5′-ATGCCGCCGTTCTGAATGA-3′; mouse *Apoe* primer, forward, 5′-GAACCGCTTCTGGGATTACCTG-3′; reverse, 5′- GCCTTTACTTCCGTCATAGTGTC-3′; mouse *Itgax* (*Cd11c*) primer, forward, 5′-CTGGATAGCCTTTCTTCTGCTG-3′; reverse, 5′-GCACACTGTGTCCGAACTCA-3′; mouse *Hbegf* primer, forward, 5′-ACCAGTGGAGAATCCCCTATAC-3′; reverse, 5′-GCCAAGACTGTAGTGTGGTCA-3′.

### Immunofluorescence study

Immunofluorescence analysis was performed as described previously [41]. In brief, mice were deeply anesthetized and perfused intracardially with PBS and 4% paraformaldehyde in PBS. The brains or spinal cords were dissected, post-fixed with the same fixative, and cryoprotected with 30% sucrose containing PBS. Twenty-micrometer-thick coronal brain sections or spinal cord transverse sections were fixed with 4% paraformaldehyde in PBS for 5 min and then permeabilized with 0.1% Triton X−100/PBS for 10 min. After an incubation in blocking solution (5% goat or donkey serum/PBS) for 1 h, sections were incubated with a combination of following antibodies: rabbit anti-Iba-1 (#019–19741, 1:500; Wako, Osaka, Japan), goat anti-AIF-1/Iba1 (#NB100-1028, 1:250; Novus Biologicals, CO, USA), mouse anti-human SOD1 (#M062-3, 1:500; MBL, Nagoya, Japan), mouse anti-AT8 (#MN1020, 1:500, Invitrogen, CA, USA), rabbit anti-Tmem119 (#ab209064, 1:500; abcam, Cambridge, UK), or mouse anti-ApoE (#sc-390925, 1:100, Santa Cruz Biotechnology, Inc, CA, USA) at 4 °C overnight. After washing with PBS, sections were incubated with fluorescent-conjugated anti-rabbit, anti-mouse, or anti-goat IgGs (1:1000; Thermo Fisher Scientific) at room temperature for 1 h. For thioflavin-S staining, after immunostaining with primary and secondary antibodies, sections were incubated with 0.02% thioflavin-S (#T1892, Sigma) at room temperature for 8 min, followed by incubation with 50% ethanol at room temperature for 1 min twice. After washing in PBS, sections were mounted on slides and analyzed with a confocal microscope (LSM700, Carl Zeiss, Oberkochen, Germany).

### Statistical analysis

For RNA sequencing data, differential expression analysis between two groups with replicates was performed with edgeR v3.24.3 [36] implemented in R v3.5.1, and *q*-value was calculated for multiple testing correction of the *p*-values with the *q*-value package v2.24.1 [42] in R. TPM (Transcripts Per Million) and CPM (Counts Per Million) values were computed with the StringTie and edgeR, respectively. A statistical comparison of the fold change values of gene expression quantities between two mutant experiments were performed with Wilcoxon’s signed rank test, followed by a multiple testing correction with the Bonferroni–Holm method [1]. The representative log fold change values of the experiments for this purpose were computed with the edgeR. For quantitative PCR, differences between two groups were analyzed by a two-tailed Student’s *t* test with the Bonferroni method.

## Results

### Altered gene expression profiles of isolated microglia in mouse models of neurodegenerative diseases

**Fig. 1:**
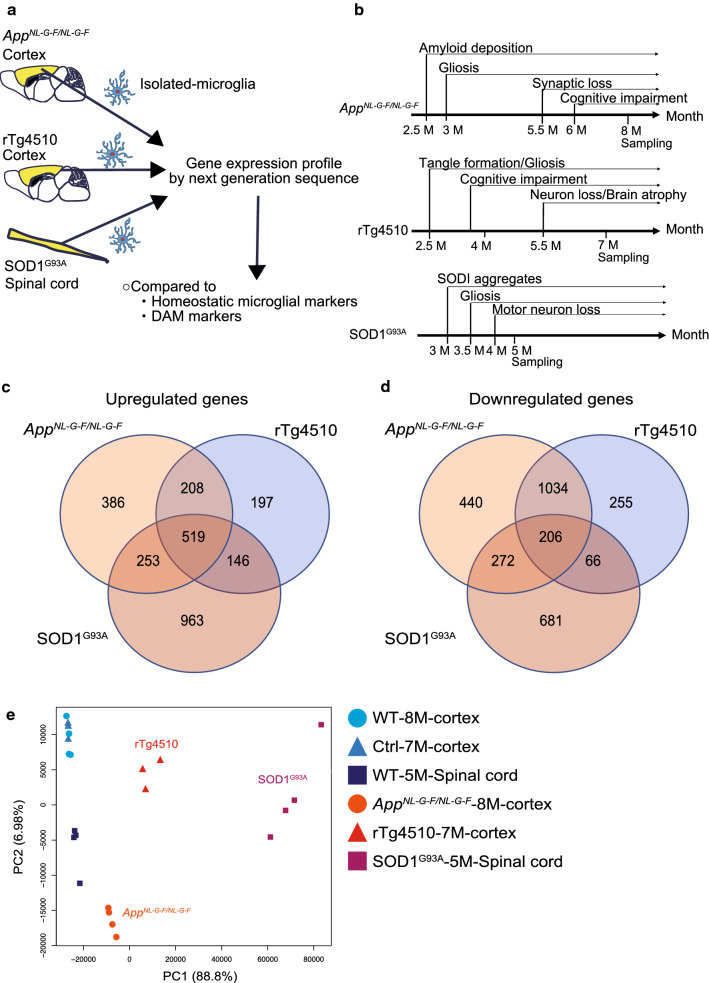
Altered gene expression profiles of microglia isolated from mouse models of neurodegenerative diseases. **a** Schematic overview of gene expression analysis of microglia isolated from mouse models of different neurodegenerative diseases. **b** Timeline of the phenotype, pathology, and sampling for each mouse model. Venn diagram displaying the numbers of significantly upregulated (**c**) and downregulated (**d**) genes among each comparison between neurodegenerative models and controls: 3318 deregulated genes (1366 upregulated and 1952 downregulated) in *App*^*NL*-*G*-*F/NL*-*G*-*F*^ microglia (n = 4 for each genotype), 2631 deregulated genes (1070 upregulated and 1561 downregulated) in rTg4510 microglia (n = 3 for each genotype), and 3106 deregulated genes (1881 upregulated and 1225 downregulated) in SOD1^G93A^ microglia (n = 4 for each genotype) (*q* < 0.05, |*FC*| > 1.5, *TPM *>* 5*). **e** Principal component analysis of gene expression profiles of microglia isolated from neurodegenerative disease models and their controls. WT, wild-type; Ctrl, control

To compare microglial phenotypes in different neurodegenerative diseases, we analyzed three representative model mice: *App*^*NL*-*G*-*F/NL*-*G*-*F*^ mice with amyloid pathology [37], rTg4510 mice with tauopathy [38], and SOD1^G93A^ mice modeling inherited ALS [12] (Fig. 1a). All mouse models exhibited accumulation of disease-causing proteins at 2.5 ~ 3 months of age followed by neuroinflammation/gliosis (Fig. 1b). Despite age-dependent typical amyloid pathology and neuroinflammation, *App*^*NL*-*G*-*F/NL*-*G*-*F*^ mice did not show neuronal loss or neurofibrillary tangles (NFTs) [37]. In contrast, rTg4510 and SOD1^G93A^ mice exhibited robust neurodegeneration. Therefore, we used the *App*^*NL*-*G*-*F/NL*-*G*-*F*^ mouse as a mild model and rTg4510 and SOD1^G93A^ mice as severe models of neurodegenerative diseases. We analyzed gene expression in microglia isolated by magnetic-activated cell sorting (MACS) from cerebral cortex of *App*^*NL*-*G*-*F/NL*-*G*-*F*^ and rTg4510 mice or lumbar spinal cord of SOD1^G93A^ mice at the middle-to-late disease stage (Fig. 1a, b).

We aimed to characterize gene expression profiles in microglia isolated from each mouse model via RNA sequencing (RNA-seq) (Fig. 1a). Analysis of expression profiles revealed 3318, 2631, and 3106 differentially expressed genes in *App*^*NL*-*G*-*F/NL*-*G*-*F*^, rTg4510, and SOD1^G93A^ microglia, respectively (Fig. 1c, d, Additional file 1: Tables S1a–c). We then performed principal component analysis (PCA) by employing normalized read counts of the genes for individual samples. Based on PCA plot, we found that the first principal component (PC1), accounting for 88.8% of the total variance, solely separated the samples into four categories comprised of the three neurogenerative and one normal phenotype (Fig. 1e). Furthermore, the four clusters were located in order of the degree of neuronal cell loss along the PC1, suggesting that gene expression profiles gradually change in accordance with the degree of neuronal cell loss.

### Decrease in expression of homeostatic microglial genes in mouse models of tauopathy and SOD1-ALS

**Fig. 2:**
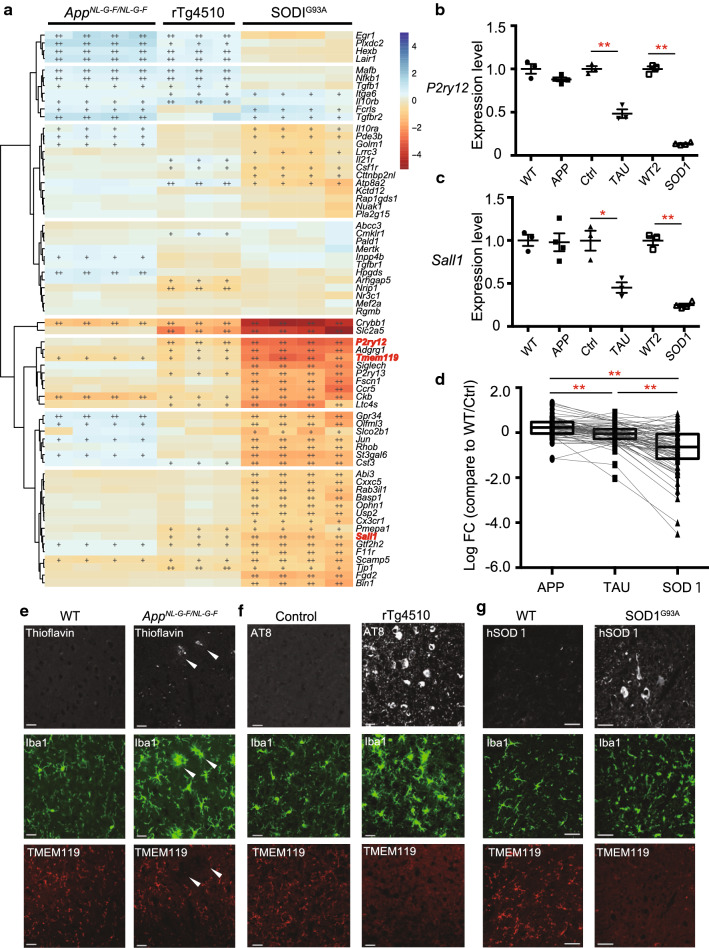
Decreases in gene expression linked to homeostatic microglia in mouse models of neurodegenerative diseases. Expression of homeostatic microglial genes analyzed by RNA sequencing (RNA-seq) and quantitative PCR (WT: n = 4 and *App*^*NL*-*G*-*F/NL*-*G*-*F*^: n = 4; Ctrl: n = 3 and rTg4510: n = 3; WT: n = 4 and SOD1^G93A^: n = 4). WT, wild-type; Ctrl, control. **a** A heat map of homeostatic microglial genes in isolated-microglia of the mouse models of neurodegenerative diseases. Colors of the individual cells denote relative expression levels of the neurodegenerative models. +: *q* < 0.05, ++: *q* < 0.001. **b**, **c** Quantitative PCR analysis to determine the expression levels of *P2ry12* and *Sall1* mRNA in isolated-microglia of each mouse model. **b** *P2ry12* expression level (*App*^*NL*-*G*-*F/NL*-*G*-*F*^: *FC* = −1.14*, p* = 0.2275; rTg4510: *FC* = −2.07, *p* = 0.00285; SOD1^G93A^*: FC* = −7.35, *p* = 1.74E−06). **c** *Sall1* expression level (*App*^*NL*-*G*-*F/NL*-*G*-*F*^: *FC* = −1.02, *p* = 1; rTg4510: *FC* = −2.21, *p* = 0.0420; SOD1^G93A^: *FC* = −4.04, *p* = 6.71E−05). Data are represented as the mean ± SEM. *p < 0.05, **p < 0.001. Bonferroni-corrected Student’s *t* test. **d** Log_2_FC values against WT/Ctrl for 68 homeostatic microglial genes in the isolated microglia from the cortex of *APP*^*NL*-*G*-*F/NL*-*G*-*F*^ and rTg4510 mice, and spinal cord of SOD1^G93A^ mice. *App*^*NL*-*G*-*F/NL*-*G*-*F*^ (median *log*_*2*_*FC* = 0.227), rTg4510 (median *log*_*2*_*FC* = −0.0504, *adj. p *=* 7.33*E−07), and SOD1^G93A^ (median *log*_*2*_*FC* = −0.633, *adj. p* = 1.61E−11) mice. Data are represented as the median with 5th and 95th percentile. *p < 0.05, **p < 0.001. Wilcoxon’s signed rank test, followed by a multiple testing correction using the Bonferroni–Holm method. **e**–**g** Representative immunofluorescent images demonstrating amyloid β (Aβ, thioflavin), Tau (AT8) or hSOD1 (white), Iba1 (green), and TMEM119 (red) in cortex of **e** WT and *App*^*NL*-*G*-*F/NL*-*G*-*F*^ mouse, **f** Ctrl and rTg4510 mouse, and **g** spinal cord of WT and SOD1^G93A^ mouse. Arrowheads indicate Aβ and Aβ-associated microglia. Scale bars: 20 µm (**e, f**) and 50 µm (**g**)

We first focused on the homeostatic microglial genes, as diseased microglia lose homeostatic functions in an apolipoprotein E (*APOE*)- and *TREM2*-dependent manner [24]. To clarify whether the loss of homeostatic microglial genes correlates with the degree of neuronal cell loss, we investigated expression levels of the 68 homeostatic genes [24], expressions of which are all enriched in mature microglia, among the three models. As mentioned earlier, *App*^*NL*-*G*-*F/NL*-*G*-*F*^ mice show neuroinflammation and an amyloid pathology without neuronal cell death, whereas rTg4510 and SOD1^G93A^ mice show robust neuroinflammation with neuronal cell death. A heat map of the 68 gene expression profiles illustrates changes in expression of homeostatic microglial genes associated with the degree of neuronal cell loss (Fig. 2a). *P2ry12* and *Sall1* exhibited a typical trend of the changes of gene expression level among the three models; a non-significant change in *App*^*NL*-*G*-*F/NL*-*G*-*F*^ microglia, but substantial reductions in rTg4510 and SOD1^G93A^ microglia (Fig. 2a–c). Moreover, the degree of reduction in expression levels of homeostatic microglial genes significantly correlated with the degree of neuronal cell loss (Fig. 2d and Additional file 1: Table S1d). Furthermore, although a loss of TMEM119 immunoreactivity, another representative homeostatic microglial gene, was restricted to amyloid plaque-associated microglia in *App*^*NL*-*G*-*F/NL*-*G*-*F*^ mice (Fig. 2e), TMEM119 immunoreactivity was decreased in microglia of rTg4510 and SOD1^G93A^ mice (Fig. 2f, g). These results indicate that decreased expression of microglial homeostatic genes correlates with the degree of neuronal cell loss.

### No association between changes in DAM genes and the degree of neuronal cell loss

**Fig. 3:**
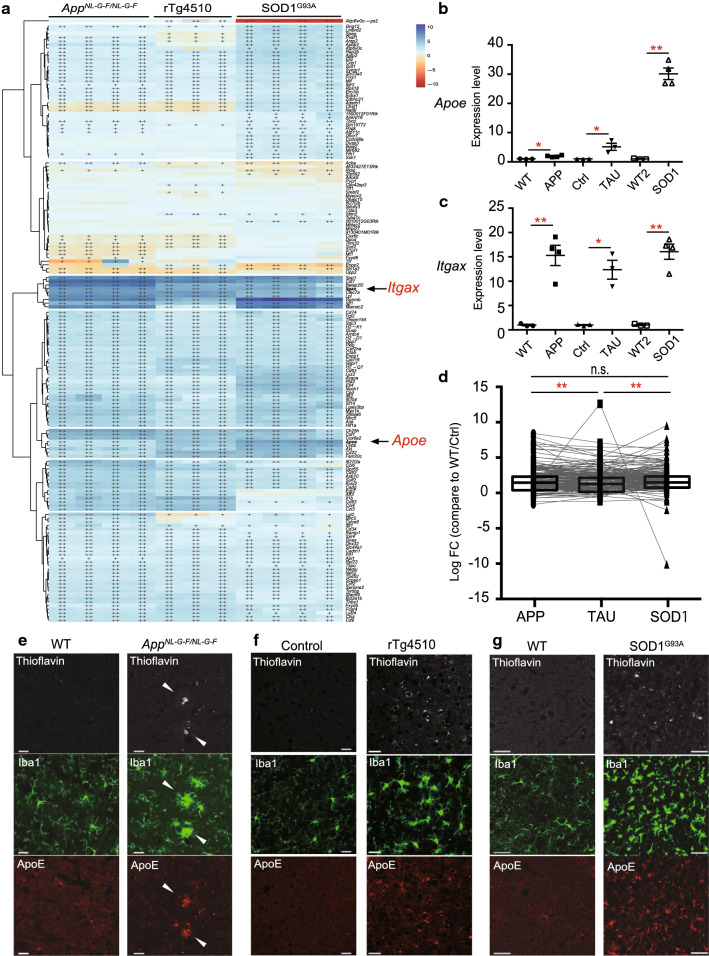
Elevated gene expression of disease-associated microglia (DAM) in mouse models of neurodegenerative diseases. DAM gene expression analyzed by RNA sequencing (RNA-Seq) and quantitative PCR (WT: n = 4 and *App*^*NL*-*G*-*F/NL*-*G*-*F*^: n = 4; Ctrl: n = 3 and rTg4510: n = 3; WT: n = 4 and SOD1^G93A^: n = 4). WT, wild-type; Ctrl, control. **a** A heat map of DAM genes in isolated-microglia. Colors indicate upregulated (blue) and downregulated genes (red) relative to each control. +: *q* < 0.05, ++: *q* < 0.001. **b**, **c** Quantitative PCR analysis to determine expression levels of *Apoe* and *Itgax* mRNA in isolated-microglia of each mouse model. **b** *Apoe* expression level (*App*^*NL*-*G*-*F/NL*-*G*-*F*^: *FC* = 1.91, *p* = 0.0126; rTg4510: *FC* = 5.51*, p* = 0.0251; SOD1^G93A^*: FC* *= 30.1, p* = 0.000177). **c** *Itgax* expression level (*App*^*NL*-*G*-*F/NL*-*G*-*F*^: *FC *= 15.3, *p* = 0.00638; rTg4510: FC =* 12.4, p* = 0.0129; SOD1^G93A^: *FC* = 16.1, *p* = 0.00138). Data are represented as the mean ± SEM. *p < 0.05, **p < 0.001. Bonferroni-corrected Student’s *t* test. **d** Log_2_FC values against WT/Ctrl for 162 DAM genes in the isolated microglia from the cortex of *APP*^*NL*-*G*-*F/NL*-*G*-*F*^ and rTg4510 mice, and spinal cord of SOD1^G93A^ mice. *App*^*NL*-*G*-*F/NL*-*G*-*F*^ (median *log*_*2*_*FC* = 1.466), rTg4510 (median *log*_*2*_*FC* = 1.275*, adj. p* = 2.12E−03), and SOD1^G93A^ (median *log*_*2*_*FC* = 1.484, *adj. p* = 1.14E−01) mice. Data are represented as the median with 5th and 95th percentile. *p < 0.05, **p < 0.001. Wilcoxon’s signed rank test, followed by a multiple testing correction using the Bonferroni–Holm method. **e**–**g** Representative immunofluorescent images demonstrating expression of Aβ, Tau, or SOD1 (thioflavin, white), Iba1 (green), and ApoE (red) in the cortex of **e** WT and *App*^*NL*-*G*-*F/NL*-*G*-*F*^ mouse, **f** Ctrl and rTg4510 mouse, and **g** the spinal cord of WT and SOD1^G93A^ mouse. Arrowheads indicate Aβ and Aβ-associated microglia. Scale bars: 20 µm (**e, f**) and 50 µm (**g**)

To examine whether DAM are common in mouse models of neurodegenerative diseases, and whether this activation is also associated with the degree of neuronal cell loss, we compared expression levels of 162 DAM genes among three mouse models (*FC* > 1.5, *q* < 0.05) [20] (Fig. 3a). A heat map shows that almost all DAM genes including *Itgax* (Fig. 3c) were uniformly upregulated in microglia of the three mouse models, while only 12 of these genes (7.41%), including *Apoe*, *Axl*, and *Cybb*, exhibited upregulation in accord with the degree of neuronal cell loss (Fig. 3a–d and Additional file 1: Table S1e). Thus, the degree of increased expression of DAM genes was not associated with the degree of neuronal cell loss (Fig. 3d and Additional file 1: Table S1e). Although upregulation of ApoE was limited in plaque-associated microglia of *App*^*NL*-*G*-*F/NL*-*G*-*F*^ mice, immunoreactivity of ApoE was increased in microglia of all three models (Fig. 3e–g). These results suggest that neurodegenerative diseases have upregulation of most DAM genes in common, but levels of expression were not correlated with the degree of neuronal cell loss.

### Differentially expressed genes for various CNS cell types in human precuneus with AD pathology

**Fig. 4:**
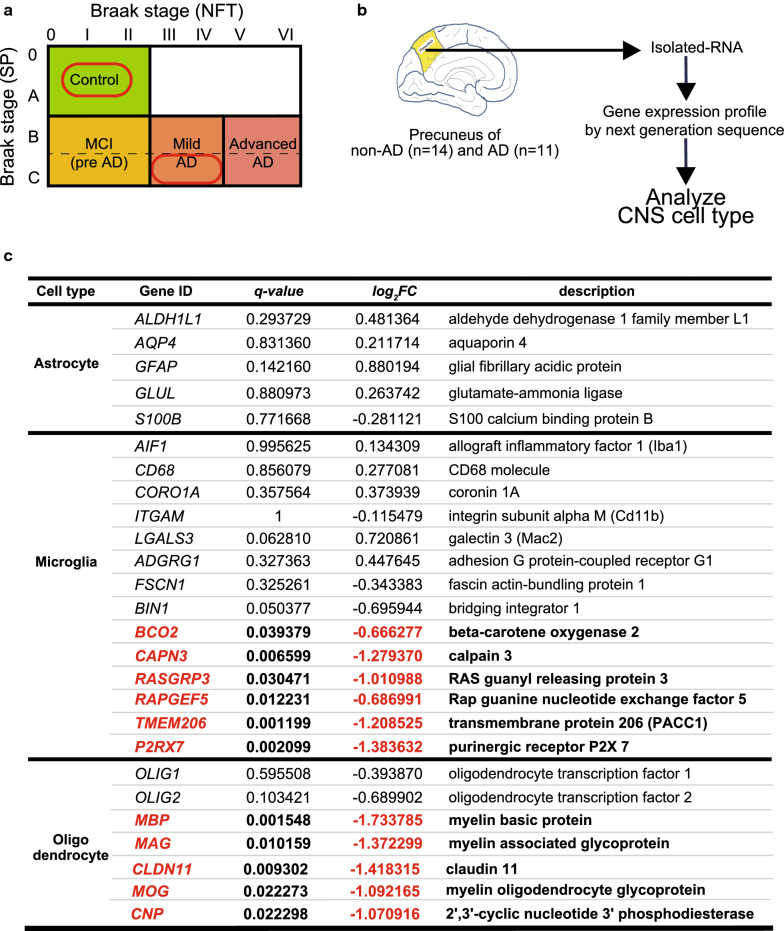
RNA sequencing reveals altered gene expression of each CNS cell-type in human precuneus with Alzheimer’s disease (AD) pathology. **a** Human brain samples were selected for analysis based on the Braak staing as follows: control brain (non-AD) defined as Braak stage (senile plaque: SP): 0–A, Braak stage (neurofibrillary tangle: NFT): 0–II; and AD brain defined as Braak stage (SP): C and Braak stage (NFT): III–IV. **b** Schematic overview of the gene expression analysis of the CNS cell-type markers in the precuneus of non-AD (n = 14) and AD (n = 11). **c** Expression of representative genes enriched in astrocytes, microglia, and oligodendrocytes in precuneus of AD brain with fold change. Downregulated genes (statistically significant, *q* < 0.05) are shown in red and bold

To uncover differentially expressed genes in human brain with an early amyloid pathology, we created a gene expression profile of precuneus derived from the individuals neuropathologically diagnosed with early AD and controls. All subjects with early AD did not have apparent family history of dementia, therefore, were recognized as sporadic cases. The precuneus is located on the medial side of the parietal cortex, which is vulnerable to early amyloid deposition in patients with AD [30, 47]. Data for Braak neuropathological stages, neuropathological diagnosis, Clinical Dementia Ratings (CDR), *APOE* genotypes, and RNA quality of human brain samples are described in Additional file 1: Table S1f. Postmortem subjects were identified and selected based on the Braak neuropathological staging as follows: 14 non-AD brains were scored 0–A for senile plaque (SP) and 0–II for NFT, and 11 AD brains were C for SP and III–IV for NFT (Fig. 4a and Additional file 1: Table S1f). We found 643 deregulated genes, consisting of 127 upregulated and 516 downregulated, in AD brains compared with the controls (|*FC*|* > 1.5, q* < 0.05) (Additional file 1: Table S1g).

In AD and other neurodegenerative diseases, neuroinflammation and neurodegeneration lead to changes in cell population of brain lesions. Therefore, we examined expression levels of representative CNS markers for each cell-type: neurons, astrocytes, microglia, and oligodendrocytes in the precuneus of AD brains and controls (Fig. 4b, Additional file 1: Table S1h). We generated a list of genes enriched in microglia under physiological conditions from previous studies [3, 5, 8] and compared gene expression profiles of AD precuneus. Although levels of the representative activation markers for microglia (*AIF1, CD68, and LGALS3*) were unchanged, expressions of *BCO2, CAPN3*, small G protein-associated genes (*RASGRP3* and *RAPGEF5)*, *PACC1* (also known as *TMEM206*), and *P2RX7* were significantly decreased in AD precuneus compared with controls (Fig. 4c). Moreover, expression of the oligodendrocyte markers, *MBP, MAG, CLDN11, MOG*, and *CNP* were significantly decreased in AD precuneus (Fig. 4c). No differences in the expression of representative neuronal and astrocytic genes were observed in the precuneus of AD brains (Fig. 4c, Additional file 1: Table S1h). These findings indicate that the early amyloid pathology induces moderate dysfunction of microglia and oligodendrocytes in AD precuneus.

### Subtle alteration of DAM gene expression in AD precuneus

**Fig. 5:**
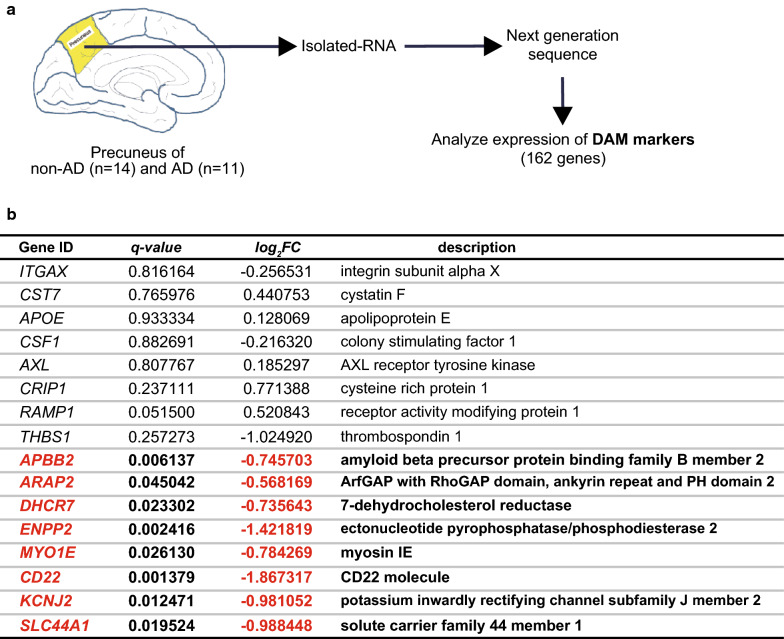
Altered expression of disease-associated microglia (DAM) genes in human precuneus of Alzheimer’s disease (AD) pathology. **a** Schematic overview of expression analysis of DAM genes in the precuneus of non-AD and AD brain. **b** List of representative DAM genes and DAM genes with significantly altered expression in the precuneus of AD brains (non-AD: n = 14 and AD: n = 11). Gene ID with description, q-value, and fold change are shown. Significantly downregulated genes (*q* < 0.05) are shown in red and bold

Reactive microgliosis with neuroinflammation is one of the neuropathological hallmarks of AD brain [9, 15, 40, 43]. Thus, we next investigated whether DAM genes are deregulated in human precuneus with early AD pathology (Fig. 5a). Among the 162 DAM genes, we found only 8 genes were deregulated in AD precuneus, and unexpectedly, all of them (*APBB2*, *ARAP2, DHCR7, ENPP2, MYO1E, CDS22, KCNJ2*, and *SLC44A1*) were downregulated (Fig. 5b). In addition, expression levels of representative DAM genes (*ITGAX, CST7, APOE, CSF1*, and *AXL*) were not altered in the precuneus of AD brain (Fig. 5b). Contrary to the upregulation of DAM genes in mouse models of AD, the expression of DAM genes was marginally altered in human sporadic AD precuneus.

### Expression levels of the genes defined as AD risk factors in microglia of mouse models of AD and human sporadic AD precuneus

**Fig. 6:**
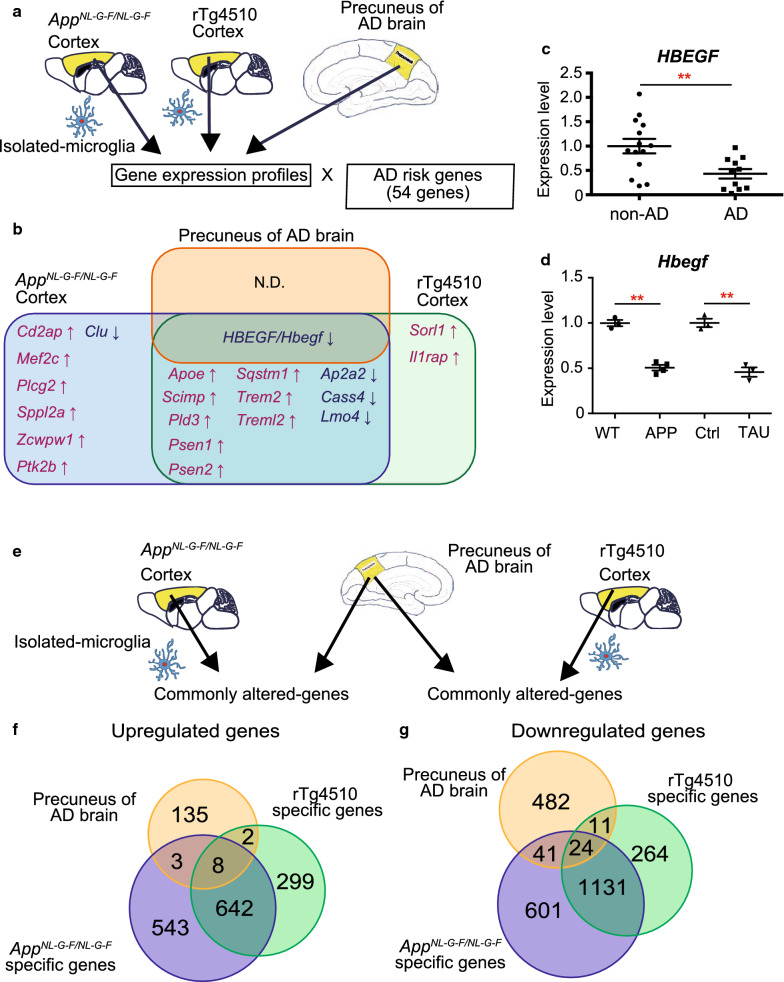
Comparative gene expression analysis of human precuneus of Alzheimer’s disease (AD) brain with microglia isolated from the AD mouse models with amyloid and Tau pathology. **a** Schematic overview of the comparative expression analysis of the genes defined as risk factors for AD in human AD precuneus and microglia isolated from *App*^*NL*-*G*-*F/NL*-*G*-*F*^ and rTg4510 mice. **b** Venn diagram displaying the differentially expressed AD risk genes among each comparison (*q* < 0.05, |*FC*| > 1.5, cut-off *TPM *>* 5*). Upregulated and downregulated genes were shown in red and black, respectively. *HBEGF/Hbegf* was the only gene commonly deregulated (AD: *log*_*2*_*FC* = −0.843, *q* = 0.00861, *App*^*NL*-*G*-*F/NL*-*G*-*F*^*: log*_*2*_*FC* = −1.710, *q* = 1.32E−23; *rTg4510: log*_*2*_*FC* = −0.738, *q* = 1.97E−05). *Cd2ap, Mef2c, Plcg2, Sppl2a, Zcwpw1, Ptk2b* and *Clu* were altered specifically in *App*^*NL*-*G*-*F/NL*-*G*-*F*^ microglia*. Sorl1 and Il1rap* were specifically deregulated in rTg4510 microglia. *Apoe, Scimp, Pld3, Psen1, Psen2, Sqstm1, Trem2, Treml2, Ap2a2, Cass4, and Lmo4l* were commonly altered in *App*^*NL*-*G*-*F/NL*-*G*-*F*^ and rTg4510 microglia. **c**, **d** Quantitative PCR analysis to determine expression level for *HBEGF/Hbegf* mRNA in human precuneus and isolated-microglia from each mouse model. **c** *HBEGF* expression level (FC = −1.83, *p* = 0.00560). **d** *Hbegf* expression level (*App*^*NL*-*G*-*F/NL*-*G*-*F*^: FC = −1.98, *p* = 0.000272; rTg4510: FC = −2.19, *p* = 0.00277). Data are represented as the mean ± SEM. *p < 0.05, **p < 0.001. Bonferroni-corrected Student’s *t* test. **c** non-AD (n = 14) and AD (n = 11). **d** WT (n = 3) and *App*^*NL*-*G*-*F/NL*-*G*-*F*^ (n = 4); Ctrl and rTg4510 (n = 3 for each genotype). WT, wild-type; Ctrl, control. **e** Schematic overview of the comparative expression analysis of microglia isolated from mice with amyloid or Tau pathology and human precuneus with early AD. **f**, **g** Venn diagrams displaying the number of significantly upregulated (**f**) and downregulated (**g**) genes among each comparison between human AD precuneus and microglia isolated from AD mice and controls: 708 deregulated genes (149 upregulated and 559 downregulated) in AD precuneus (non-AD: n = 14 and AD: n = 11; *q* < 0.05, *|FC| *>* 1.2*); 2993 deregulated genes (1196 upregulated and 1797 downregulated) in *App*^*NL*-*G*-*F/NL*-*G*-*F*^ microglia (n = 4 for each genotype); and 2381 deregulated genes (951 upregulated and 1430 downregulated) in rTg4510 microglia (n = 3 for each genotype) (*q* < 0.05, |*FC*| > 1.5, *TPM* > 5)

We evaluated expression levels of 54 genes [5, 13], which were previously defined as risk factors for AD by genome-wide association study, in human sporadic AD brains and isolated microglia from *App*^*NL*-*G*-*F/NL*-*G*-*F*^ and rTg4510 mice (Fig. 6a and Additional file 1: Table S1i). The previous study identified *GRIN2B*, *INPP5D* and *PSEN1* as significantly altered genes in the temporal or frontal cortex of AD brain [5]. We focused on those risk genes, first confirming their expressions in microglia by the transcriptome database [48], and then examined their expression levels in human AD precuneus and AD model mice (*cut*-*off TPM* > 5*, q* < 0.05; Fig. 6b). We found no upregulated genes, but the expression level of *HBEGF* (*log*_2_*FC* = −0.843, *q* = 0.00861) was significantly decreased in human AD precuneus (Fig. 6b). Further, of 54 risk genes with significantly altered expression, we found 19 and 14 deregulated genes in cortical microglia of *App*^*NL*-*G*-*F/NL*-*G*-*F*^ and rTg4510 mice, respectively. Comparison of AD risk genes among the three groups (human AD, *App*^*NL*-*G*-*F/NL*-*G*-*F*^ *and* rTg4510) revealed that 12 genes (*Apoe, Scimp, Pld3, Psen1, Psen2, Sqstm1, Trem2, Treml2, Ap2a2*, *Cass4, Lmo4 and Hbegf*) were commonly altered in *App*^*NL*-*G*-*F/NL*-*G*-*F*^ *and* rTg4510 cortical microglia, and only one gene (*HBEGF/Hbegf*) was significantly decreased in AD precuneus as well as *App*^*NL*-*G*-*F/NL*-*G*-*F*^ *and* rTg4510 cortical microglia (Fig. 6b and Additional file 1: Table S1i). In addition, *HBEGF/Hbegf* expression levels in human precuneus and mouse microglia of either models were validated by quantitative PCR (Fig. 6c, d), and were consistent with the RNA-seq data (Fig. 6a, b and Additional file 1: Table S1i). These results indicate that expression changes of AD risk genes were robust in microglia of the mouse models of AD, but minor in the precuneus of individuals with early AD pathology.

### Profiling of commonly altered genes in human sporadic AD precuneus and microglia of the mouse models of AD

To understand the neuroinflammatory nature of early amyloid pathology in AD, we generated expression profiles of commonly altered genes in the precuneus of human sporadic AD brain and cortical microglia isolated from *App*^*NL*-*G*-*F/NL*-*G*-*F*^ and/or rTg4510 mice (Fig. 6e). We compared gene profiles of AD precunei (|*FC*|>* 1.2, q* < 0.05) and deregulated genes of cortical microglia of *App*^*NL*-*G*-*F/NL*-*G*-*F*^ or rTg4510 mice (|*FC*| > 1.5, *q* < 0.05*, cut*-*off TPM* > 5). We found that deregulated 32 genes, consisting of 8 upregulated and 24 downregulated, were common among AD precuneus, *App*^*NL*-*G*-*F/NL*-*G*-*F*^ and rTg4510 (Fig. 6f, g, Additional file 1: Tables S1j and k). The eight upregulated genes include a chemokine (*CXCL10*), and interferon-induced genes (*STAT1, IFIT3, ISG15*), indicating neuroinflammatory changes in AD precuneus and AD mice (Table S1j). In human precuneus of AD brains and *App*^*NL*-*G*-*F/NL*-*G*-*F*^ mice, we found 44 deregulated genes (3 upregulated and 41 downregulated), including *TNFSF10 and SLC6A8*, whereas in AD precuneus and rTg4510 mice, we found 13 deregulated genes (2 upregulated and 11 downregulated) including *PTPRD*. These results suggest that some deregulated genes are common between microglia of different AD mouse models and human sporadic AD precuneus, and that *App*^*NL*-*G*-*F/NL*-*G*-*F*^ and rTg4510 cortical microglia may represent different neuroinflammatory aspects relevant to AD pathologies.

## Discussion

Our gene expression analysis of isolated microglia from mice of three neurodegenerative disease models including AD revealed that downregulation of homeostatic microglial genes and robust upregulation of DAM genes. More importantly, the extent of downregulation of homeostatic microglial genes was correlated with the degree of neuronal cell loss. In addition, a gene expression profile of human precuneus of early AD pathology shows reduced expression of some microglial homeostatic genes without induction of DAM genes, suggesting that the microglial signature in human early AD has limited similarity to that found in AD mice.

Previous studies reported a loss of homeostatic microglial genes in neurodegenerative diseases such as AD, ALS, and multiple sclerosis [20, 24, 50]. In contrast to their findings, we found that rTg4510 and SOD1^G93A^ mice exhibited more robust downregulation in homeostatic microglial genes than that of *App*^*NL*-*G*-*F/NL*-*G*-*F*^ mice, which exhibit neuroinflammation without neuronal loss (Fig. 2). In addition, rTg4510 mice show brain atrophy because of neurodegeneration, and SOD1^G93A^ mice exhibit fatal motor neurodegeneration. Of particular interest, changes in homeostatic microglial genes include *P2ry12*, *Sall1*, and *Tmem119* (Fig. 2a–d). P2RY12 regulates microglial activation via extracellular nucleotides [3, 14]. SALL1 inhibits a reactive microglia phenotype and promotes a physiological surveilling phenotype [17], and TMEM119 is expressed in microglia-derived cells and is absent in macrophages [3, 46]. Although a loss of Tmem119 was limited to Aβ plaque-associated microglia in *App*^*NL*-*G*-*F/NL*-*G*-*F*^ mice, more robust decreases in Tmem119 were observed in rTg4510 and SOD1^G93A^ microglia. Although there is a possibility that a loss of homeostatic microglial genes might be attributed to overexpression of transgenes, our result indicates that a loss of unique microglial homeostatic genes correlates with severity of neurodegeneration, and that decreased homeostatic microglial markers may be one of the hallmarks of progressive neuronal loss.

Consistent with the previous studies [3, 20, 24], we found that DAM genes were uniformly upregulated in all three neurodegenerative mouse models. However, we also found that upregulation of most DAM genes was not correlated with the degree of neuronal cell loss (Fig. 3a, c, d). As a rare exception, *Apoe* was correlated with the degree of neuronal cell loss (Fig. 3b), and the change in *Apoe* expression was negatively associated with altered expression of homeostatic microglial genes (Fig. 2). The ApoE4 triggers induction of DAM with impaired homeostatic functions and is responsible for driving neurodegeneration [24, 39]. This finding indicates that ApoE signaling may be involved in the loss of homeostatic microglial function. Combined with previous studies, our findings suggest that most DAM genes do not directly accelerate progression of neurodegeneration, but a few DAM genes, such as *APOE* may be involved in severity of neurodegeneration. Since the relationship between *APOE* genotypes and expressions of DAM and homeostatic microglial genes cannot be determined due to the limitation of *APOE e4* carriers in our study, further studies are required to clarify the potential role of *APOE e4* in human microglial phenotypes.

Despite its importance as a vulnerable region of amyloid deposition in early AD, gene expression in the precuneus of patients with AD has not been well explored in the past. Our study provides the first comprehensive gene expression profile of precuneus at the early stage of AD pathology (Braak NFT stage III–IV). Although the slight elevation of astrocytic and microglia activation markers in early AD precuneus was not statistically significant, the upregulation of chemokine and proinflammatory genes (*CXCL10, STAT1, ISG15*, and *ITIF3*) indicates neuroinflammatory changes in early AD precuneus. We also found downregulation of several microglial markers and DAM genes in early AD precuneus (Figs. 4, 5). In particular, the unaltered state of most DAM genes in human AD precuneus was in striking contrast with the data from AD mouse models. One possible interpretation is that the expression of genes of human AD and mouse models of AD is discordant, as demonstrated by a study using 5XFAD mice [49]. Another possibility is that microglial function may be suppressed at the early stage of amyloid pathology. A recent study pointed out the low sensitivity of single nucleus RNA-seq to detect DAM genes in human postmortem brains [45]. This low sensitivity may be attributed to redistribution of DAM mRNAs to the cytosol or instability of DAM mRNAs. As our study is based on the RNA-seq of whole brain tissues, cytosolic mRNAs can be detected, therefore, the latter possibility namely, instability of DAM mRNAs in human postmortem brain, remains. Further comparative gene expression analyses using the brain samples with early and advanced neuropathology will enable a conclusion regarding the significance of homeostatic microglia and DAM in human AD.

Another striking finding was the robust downregulation of genes linked to oligodendrocytes (*MBP, MAG, CLDN11, MOG,* and *CNP*) in early AD precuneus. In recent years, increasing evidence suggests that white matter abnormalities are also an important component of AD [28], and oligodendrocyte abnormalities in advanced AD brain were also reported by a recent transcriptomic study [49]. Compared with the microglia-mediated neuroinflammation, the role of oligodendrocytes in AD has been underestimated. Our data of the deregulation of oligodendrocytes and microglia in early AD precuneus suggest that both microglia and oligodendrocyte dysfunction may play a role in development of early AD pathology.

Although many risk genes for AD have been linked to microglia functions [13], we found downregulation of *HBEGF/Hbegf* was the only common change among the AD risk genes in human precuneus of early AD and AD model mice. A previous study reported an association between the single nucleotide polymorphism rs77493189 in *HBEGF* and late-onset AD [25]. Moreover, forebrain-specific *Hbegf* knockout mice exhibited neurotrophic and growth factor imbalances as well as impaired memory function and synaptic plasticity [29]. In this study, we performed comparative analysis of the gene expression profiles of sporadic AD precuneus and microglia isolated from familial AD models. Therefore, a small number of risk genes commonly deregulated in human precuneus and mouse microglia may be attributed to the different etiology between sporadic and familial AD. Despite of the limitation in our study, our results suggest that supplementation of HBEGF may be a viable therapeutic target for AD.

Comparative analysis of the gene expression profiles of AD precuneus and microglia isolated from *App*^*NL*-*G*-*F/NL*-*G*-*F*^ and rTg4510 mouse cortices revealed that the number of commonly altered genes with AD precuneus were greater in *App*^*NL*-*G*-*F/NL*-*G*-*F*^ microglia (76 genes) compared with rTg4510 microglia (45 genes). *App*^*NL*-*G*-*F/NL*-*G*-*F*^ mice specifically shared 44 deregulated genes with AD precuneus, and *TNFSF10*, one of the upregulated genes, is implicated in neuroinflammation and Aβ accumulation [4]. In contrast, *SLC6A8* is one of the 41 downregulated genes, and it mediates inflammation by regulating creatine uptake [19]. We also found that *PTPRD* was decreased in both AD precuneus and rTg4510 mice; an association was reported between the rs560380 polymorphism in *PTPRD* and NFT burden [7]. These results suggest that *App*^*NL*-*G*-*F/NL*-*G*-*F*^ and rTg4510 cortical microglia represent different aspects of AD pathologies.

## Conclusions

Microglial gene signature in mice revealed that a loss of homeostatic microglia function is associated with the degree of neuronal cell loss. In humans, our evaluation of the precuneus of early AD pathology also suggests a loss of microglia and oligodendrocyte function induced by early amyloid pathology. Results from the present study indicate a correlation between glial phenotypes and severity of neurodegeneration, and also provide important resources to better understand the role of glial dysfunction in AD progression.

## Supplementary Information

**Additional file 1: Table S1** Assembled multiple supplementary tables containing data for RNA sequencing and clinical and neuropathological data of human subjects. **Table S1a**. A list of differentially expressed genes in isolated-microglia from *App*^*NL*-*G*-*F/NL*-*G*-*F*^ mice. **Table S1b**. A list of differentially expressed genes in isolated-microglia from rTg4510 mice. **Table S1c**. A list of differentially expressed genes in isolated-microglia from SOD1^G93A^ mice. **Table S1d**. Changes in homeostatic microglial markers in each mouse model of neurodegenerative disease. **Table S1e**. Changes in DAM markers in each mouse model of neurodegenerative disease. **Table S1f**. Clinical and neuropathological data of human subjects. **Table S1g**. A list of differentially expressed genes in the precuneus of AD brain. **Table S1h**. A list of differentially expressed genes enriched in neurons in the precuneus of AD brain. **Table S1i**. Changes in the genes defined as risk factors for AD in the AD precuneus and microglia isolated from *App*^*NL*-*G*-*F/NL*-*G*-*F*^ and rTg4510 mice. **Table S1j**. A list of commonly upregulated genes in the precuneus of AD brain and isolated-microglia from *App*^*NL*-*G*-*F/NL*-*G*-*F*^ mice and/or rTg4510 mice. **Table S1k**. A list of commonly downregulated genes in the precuneus of AD brain and isolated-microglia from *App*^*NL*-*G*-*F/NL*-*G*-*F*^ mice and/or rTg4510 mice.

## Abbreviations

Aβ: amyloid β
AD: Alzheimer’s disease
ALS: amyotrophic lateral sclerosis
Ap2a2: adaptor related protein complex 2 subunit alpha 2
APOE: apolipoprotein E
App: amyloid precursor protein
AXL: AXL receptor tyrosine kinase
Cass4: cas scaffold protein family member 4
CPM: counts per million
CXCL10: C-X-C motif chemokine ligand 10
CYBB: cytochrome b-245 beta chain
DAM: disease associated microglia
Hbegf: heparin binding EGF like growth factor
IFIT3: interferon induced protein with tetratricopeptide repeats 3
ISG15: ISG15 ubiquitin-like modifier
ITGAX: integrin subunit alpha X
LMO4: LIM domain only 4
NFT: neurofibrillary tangle
PET: positron emission tomography
P2RY12: purinergic receptor P2Y12
SP: senile plaque
PLD3: phospholipase D family member 3
PSEN1: presenilin 1
PSEN2: presenilin 2
SALL1: spalt like transcription factor 1
Scimp: SLP adaptor and CSK interacting membrane protein
SLC6A8: solute carrier family 6 member 8
SOD: superoxide dismutase
Sqstm1: sequestosome 1
STAT1: signal transducer and activator of transcription 1
TMEM119: transmembrane protein 119
TNFSF10: TNF superfamily member 10
TPM: transcripts per million
Trem2: triggering receptor expressed on myeloid cells 2
Treml2: triggering receptor expressed on myeloid cells like 2

## Availability of data and materials

Data, material and software information supporting the conclusions of this article is included within the article and its additional files. The raw RNA-seq datasets analyzed in the current study are available from the corresponding author on reasonable request, and will be deposited to a public data repository.
